# DNMT3A mutation promotes leukemia development through NAM-NAD metabolic reprogramming

**DOI:** 10.1186/s12967-023-04323-z

**Published:** 2023-07-18

**Authors:** Xuejiao Yang, Xiao Wang, Ying Yang, Zhiyang Li, Yunshuo Chen, Siqi Shang, Yueying Wang

**Affiliations:** 1 Shanghai Institute of Hematology, State Key Laboratory of Medical Genomics, National Research Center for Translational Medicine at Shanghai, Ruijin Hospital, Shanghai Jiao Tong University School of Medicine, Shanghai, 200025 China; 2 Department of Neurosurgery, Renmin Hospital of Wuhan University, Wuhan, 430000 Hubei China

**Keywords:** DNMT3A mutation, Metabolic reprogramming, NAM-NAD metabolism, NAMPT, AML

## Abstract

**Background:**

DNA methyltransferase 3A (DNMT3A) is frequently mutated in acute myeloid leukemia (AML) with Arg882His (R882H) as the hotspot mutation. It has been reported that DNMT3A mutation plays a key role in leukemogenesis through hypomethylation of some target genes associated with cell growth and differentiation. In this study, we investigated the function of DNMT3A R882H in the malignant progression of AML by regulating metabolic reprogramming.

**Methods:**

Ultra-High Performance Liquid Chromatography–High Resolution Tandem Mass Spectrometry (UHPLC-HRMS/MS) was used to detect metabolites in the serum of mice harboring Dnmt3a R878H mutation and the wild-type Dnmt3a. Methylated DNA Immunoprecipitation Sequencing (MeDIP-seq) and RNA sequencing (RNA-seq) were used to analyze the levels of DNA methylation and mRNA expression of genes in mouse Gr1^+^ bone marrow cells respectively. The TCGA and GO databases were used to analyze the differential genes between human samples carrying the DNMT3A R882 mutation and the wild-type DNMT3A. Co-immunoprecipitation and immunoblotting were used to illustrate the binding levels of Cyclins-CDKs and CDK inhibitors including CDKN1A and CDKN1B. Flow cytometry was used to analyze the cell differentiation, division, apoptosis and cell cycle. The effect of NAMPT inhibition on leukemia was evaluated by using in vivo fluorescence imaging in NOG mouse model bearing OCI-AML3 cells.

**Results:**

DNMT3A mutation caused high expression of nicotinamide phosphoribosyltransferase (NAMPT), a key enzyme in the nicotinamide adenine dinucleotide (NAD) salvage synthetic pathway, through DNA hypomethylation, and finally led to abnormal nicotinamide (NAM) metabolism and NAD synthesis. The NAM-NAD metabolic abnormalities caused accelerated cell cycle progression. Inhibition of NAMPT can reduce the binding degree between Cyclins-CDKs, and increase the binding interaction of the CDK inhibitors with Cyclins-CDKs complexes. Moreover, cells with high expression of NAMPT were more sensitive to the NAMPT inhibitor FK866 with a lower IC50. The inhibition of NAMPT can remarkably extend the survival time of tumor-bearing mice and reduce the infiltration of tumor cells.

**Conclusions:**

Taken together, our data showed that DNMT3A mutation caused NAMPT overexpression to induce the reprogramming of NAM-NAD metabolism and contribute to abnormal proliferation, which provided a potential direction for targeted therapy at the metabolic level in AML with DNMT3A mutation.

**Supplementary Information:**

The online version contains supplementary material available at 10.1186/s12967-023-04323-z.

## Introduction

Tumors are fundamentally characterized by self-sufficient growth signals, unlimited replication capacity, sustained angiogenesis and tissue metastasis, and resistance to cell death. In recent years, an increasing number of studies have shown that metabolic pathways are reprogrammed to meet the material and energy requirements of tumor occurrence and development during tumor evolution [1, 2]. The gut microbe *F. nucleatum* could promote carcinogenesis through the augmentation of glucose metabolism in colorectal cancer [3]. And there is a close relationship between genetic abnormalities and metabolic reprogramming, for example, activation of oncogenes and inactivation of tumor suppressor genes can promote metabolic reprogramming [4, 5]. Oncogenic transcription factors, such as MYC and hypoxia-inducible factor (HIF), can trigger nutrient acquisition by upregulating the expression of genes implicated in the uptake and metabolism of vital nutrients such as glucose and glutamine [6].

The interplay between metabolic reprogramming and epigenetics is also a crucial aspect of cancer progression. The regulation of cancer metabolism is controlled by epigenetic mechanisms at the transcriptional and post-transcriptional levels [7]. The involvement of epigenetic modifications, chromatin remodeling, and noncoding RNAs in cancer metabolic regulatory networks further contributes to malignant transformation of tumors [8, 9]. Perturbations in metabolic pathways and changes in intermediate metabolites lead to changes in the epigenetic landscape by modulating the function of epigenetic enzymes, thereby conveying information about intracellular metabolic conditions to the nucleus [10].

DNA methyltransferase 3A (DNMT3A) is one of the important genes related to epigenetic modification, which undertakes the modification of DNA de novo methylation. It is a key gene regulating the differentiation and proliferation of hematopoietic stem cells (HSC), and playing an important role in the occurrence and development of hematological tumors [11, 12]. DNMT3A mutations occur in 18%-23% of acute myeloid leukemia (AML) patients, and the mutation hotspot is located at arginine 882 position (R882), which results in decreased methyltransferase activity [13]. It is worth noting that since the DNMT3A mutation itself is a function-inactivating mutation, the targeted therapy of the DNMT3A mutation should be more inclined to select downstream regulatory genes as targets.

In recent years, many studies in tumor and non-tumor fields have paid attention to the role of DNMT3A in metabolic regulation. In cardiomyocytes, DNMT3A knockout mediates abnormal activation of the receptor of PPARγ that is the key regulator of glucose/lipid metabolism through the accumulation of lipid vacuoles. Furthermore, DNMT3A knockout can affect the HIF-1α stability to mediate the impaired glucose metabolism and the reduced expression of glycolytic enzymes, and ultimately make the heart more sensitive to metabolic stress such as serum withdrawal and food restriction [14]. In AML cell lines K562 and SKM1 that are introduced into the DNMT3A R882H mutation, the mutant DNMT3A upregulates some genes critical for glutathione synthesis, especially SLC7A11 (cysteine/glutamate transporter), resulting in a marked increase in intracellular GSH levels, thereby promoting cell proliferation. Moreover, this mutant clone is resistant to chemotherapy and SLC7A11 inhibitors, targeting the GSH synthesis pathway can improve the therapeutic effect on AML patients with the DNMT3A mutation [15].

Our team has carried out a series of research on the metabolomics of AML, and found that the overall metabolic profile of AML patients changed significantly, including enhanced glycolysis, enhanced tricarboxylic acid cycle metabolism, and up-regulated 2-hydroxyglutarate (2-HG) level, all of which were closely related to poor prognosis of AML patients [16–18]. Compared with normal cells, AML cells have more active glycolytic metabolism, accompanied by a higher demand for nicotinamide adenine dinucleotide (NAD), an important coenzyme of the glycolytic pathway. NAD is an essential metabolite required for a variety of biological processes and closely related to aging, tumorigenesis, and immune checkpoint regulation. The dependence of tumors on different pathways of NAD synthesis differs according to gene amplification and epigenetic remodeling of tissue lineage [19, 20]. Nicotinamide phosphoribosyltransferase (NAMPT), an enzyme that converts nicotinamide (NAM) to NAM mononucleotide (NMN) and finally converted to NAD, controlling the salvage synthetic pathway of NAD. NAMPT is overexpressed in colorectal cancer, gastric cancer and glioma, which is associated with tumor growth, metastasis, invasion and cell dedifferentiation, suggesting that NAMPT could be a potential anticancer target [21–28]. Elevated NAM metabolism due to high NAMPT expression in the leukemia stem cells (LSCs) of relapsed AML is also the mechanistic basis of venetoclax/azacytidine resistance as well as of the metabolic vulnerability of relapsed/refractory LSCs [29].

To date, there have been no in vivo studies of the regulation of metabolic reprogramming by the DNMT3A mutation. We established a Dnmt3a R878H (homologous to human DNMT3A R882H) conditional knock in mouse model, and found that the Dnmt3a mutation promoted mTOR gene expression through hypomethylation regulation to induce AML, and mTOR inhibitors prolonged survival of AML mice [30]. However, whether and how the DNMT3A mutation promotes the malignant progression of AML through metabolic reprogramming remain unclear.

The purpose of this study is to explore whether the DNMT3A mutation could cause the metabolic reprogramming of leukemia cells, and to reveal the underlying modulation mechanism and the effects of targeted intervention on the key factors. In conclusion, our study demonstrated that the high expression of NAMPT caused by the DNMT3A mutation led to abnormal NAM-NAD metabolism and conferred sensitivity to NAMPT inhibition. Knockdown and inhibition of NAMPT can affect the formation of CDK2-CCNE2 and CDK4-CCND3 complexes, and induce cell cycle arrest in the G1/S phase. Moreover, the inhibition of NAMPT can induce apoptosis and partial differentiation of AML cells carrying the DNMT3A mutation.

## Materials and methods

### Flow cytometry

Cells were harvested and stained using antibodies for the cell markers CD11b (Biolegend), CD14 (Biolegend) to detect the cell differentiation. The mixture was incubated in 100 μl PBS supplemented with 2% FBS in darkness for suitable length of time at 4 °C before detection. An isotype-matched antibody served as a negative control. Cells were stained with CFSE and cultured for 48–72 h before measuring the intensity of fluorescence to detect cell division (Beyotime). For the apoptosis assay, the cells were stained using the APC Annexin V Apoptosis Detection Kit with Propidium Iodide (PI) (Biolegend) according to the manufacturer's instructions and then detected by flow cytometry analysis. Flow cytometry was performed on eight-laser cytometers (BD). All data were analyzed using FlowJo software.

### Statistical analysis

Part of the data was analyzed with R, and the specific methods will be described later. Other data analysis was performed with Graphpad prism 8.0, and data was expressed as a mean ± SD. The statistical significance between groups was assessed using a Student’s T-test. For all in vivo survival experiments, Kaplan–Meier estimates of the survival function were calculated for each group and differences assessed using the log-rank test. P value < 0.05 was regarded as statistically significant.

### Cell culture

Cell lines including OCI-AML3, U937, U937^VEC^, U937^WT^, and U937^MUT^ were cultured in RPMI 1640 medium (Gibco) containing 10% fetal bovine serum (Gibco), 100 μg/ml streptomycin, and 100 IU/ml penicillin. All the above cell lines were maintained at 37 °C under 5% CO2. Moreover, cell samples were ruled out mycoplasma contamination.

### Cell cycle analysis

Cell cycle distribution was analyzed using cell cycle staining solution. The cell samples were washed with pre-cooled PBS, and then gently dropped into 75% cold ethanol to fix the samples. Finally, the cells were washed with PBS and resuspended. After adding RNaseA (50 μg/ml) and incubated at 37 °C for half an hour, the PI (50 ug/ml) was added and incubated at room temperature under darkness for 15 min before flow cytometry analysis. Data were analyzed using FlowJo software (Version 10.4).

### Cell viability assay

For cytotoxicity analysis, 10,000 cells of each group were seeded in a 96-well plate. After different time and concentration drug treatments, 10 μl CCK-8 (YiSheng) was added to each well and cultured in a cell incubator for 1 h. Finally, the absorbance was measured at 450 nm by a micro-plate reader (Tecan Infinite 200). There were 4 replicate wells in each group and three replicates were performed.

### Metabolite detection

Serum and cell metabolites were detected by coupling Ultimate 3000 Ultra-High Performance Liquid Chromatograph (UHPLC) and Q Exative Quadrupole-Electrostatic Field Orbitrap Mass Spectrometer from Thermo Fisher. The raw data were normalized for principal component analysis (PCA). Orthogonal Partial Least Squares-Discriminant Analysis was selected to characterize the differences between groups. Intracellular NAD+/NADH levels were detected using NAD+/NADH detection Kit (WST-8 method) (Beyotime). Intracellular adenosine levels were detected using adenosine assay Kit (Abcam).

### Western blot analysis

Western blot assays were performed to detect protein expression. Primary antibodies were used at a dilution of 1:1000 and incubated overnight at 4 °C. The anti-NAMPT, anti-CD38, anti-DNMT3A, anti-Caspase8, anti-Caspase9, and anti-Sirt6 antibodies were purchased from Abcam. The anti-Caspase3 and anti-Caspase7 antibodies were purchased from Santa Cruz (USA). Other antibodies were purchased from Cell Signaling Technology. Goat anti-rabbit or mouse IgG-HRP was used as a secondary antibody at 1:3000 dilution. Detection was performed by using ECL western blotting substrate.

### Quantitative reverse transcription-polymerase chain reaction (Q-RT-PCR)

Total cellular RNA was extracted using the spin-column-based EZ-Press RNA purification kit and cDNA was obtained by reverse transcription according to the instructions. Q-RT-PCR was performed using an ABI 7500 real-time PCR machine (Applied Biosystems) based on Real Master Mix (YiSheng). β-Actin was used as the endogenous control gene. The 2^−△△Ct^ method was used to obtain the relative expression levels of genes in the form of fold variation. Additional file 1: Table S1 lists the primer sequences.

### Animal studies

The NOG mice were purchased from Charles River. The mice were randomly divided into 3 groups for the following experiments (n = 6 for each group). On the first day, two groups of mice were injected with OCI-AML3 and the remaining group was injected with shNAMPT-OCI-AML3 (3 × 10^6^ cells per mouse). After 3 days, tumor proliferation in mice was observed by fluorescence imaging. For the experimental group, mice were treated daily with the NAMPT inhibitor FK866 (10 mg/kg/day) starting from day 4. The IVIS spectral imaging system was used to measure the bioluminescence signal twice a week. All animal studies were performed in accordance with ethical standards and were approved by the Committee on Animal Care and Use for Research at Shanghai Jiao Tong University School of Medicine, which are consistent with the guidelines of the Association for Assessment and Accreditation of Laboratory Animal Care international.

## Results

### Dnmt3a^R878H/WT^ mouse model exhibits abnormal NAM metabolism via regulating the expression of Nampt

**Fig. 1:**
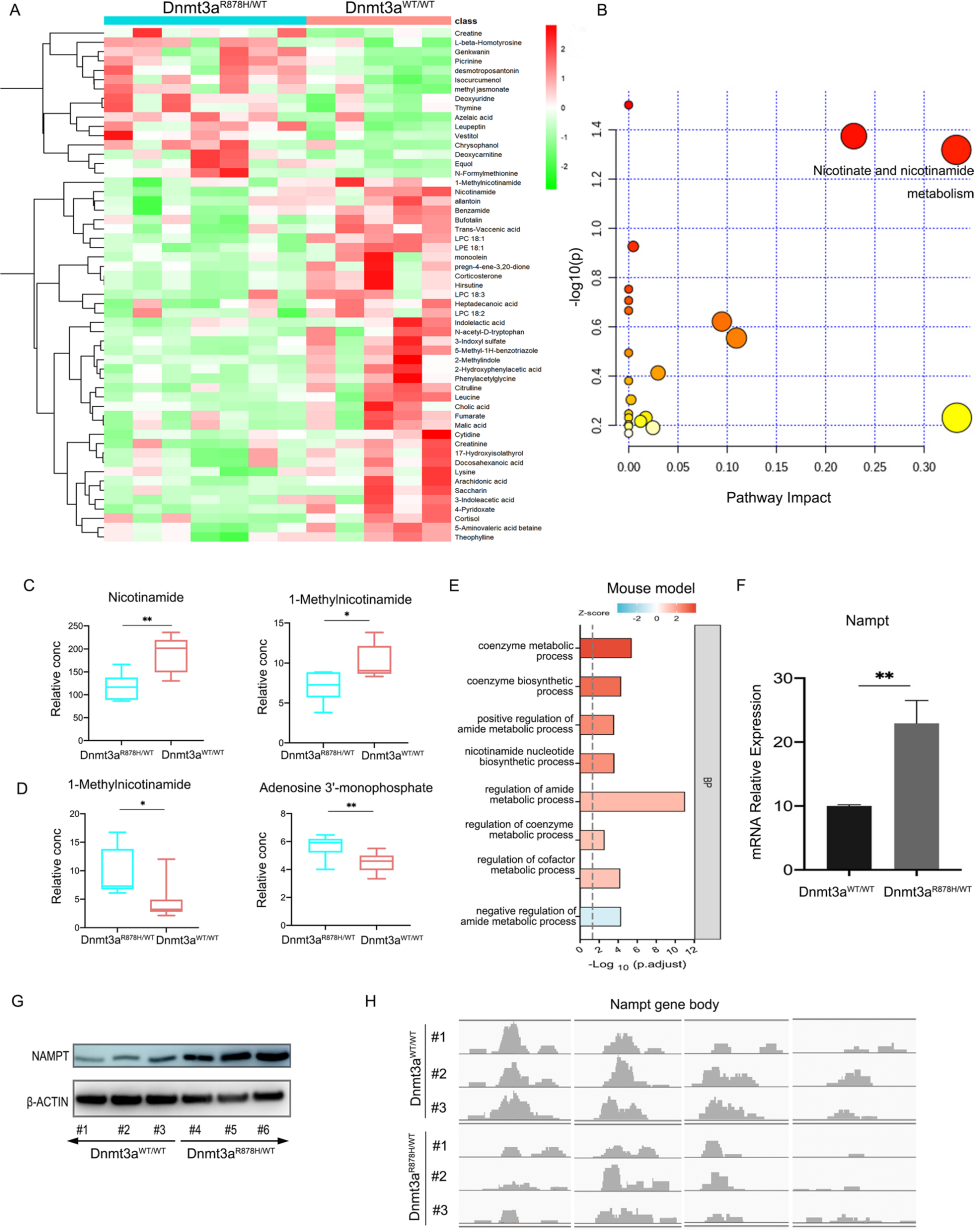
The Dnmt3a R878H mutation causes abnormal NAM metabolism by regulating the expression of Nampt. **A** UHPLC-HRMS/MS detection results of the serum of the Dnmt3a^R878H/WT^ mice and the Dnmt3a^WT/WT^ mice. **B** KEGG enrichment analysis of the 55 differential metabolites between the two groups. -Log(p) indicates the correlation between the metabolic pathway and the difference between the Dnmt3a^R878H/WT^ group and the Dnmt3a^WT/WT^ group. The redder the color of the circle, the greater the correlation. Pathway impact value is based on the pathway topology analysis, indicating the importance of the pathway for this difference to occur. The larger the circle, the greater the importance. **C** Levels of NAM and 1-methylnicotinamide in serum of the two groups. **D** Levels of 1-methylnicotinamide and Adenosine 3'-monophosphate in Gr1^+^ cells of the two groups. **E** GO enrichment analysis of the differential genes in Gr1^+^ cells of the Dnmt3a^R878H/WT^ mice vs. the Dnmt3a^WT/WT^ mice. **F**, **G** The Nampt transcriptional and translational levels of Gr1^+^ cells in the Dnmt3a^R878H/WT^ and Dnmt3a^WT/WT^ mice. **H** MeDIP-seq analysis of the Nampt gene showed a local hypomethylation pattern in the gene body region of Gr1^+^ cells from the Dnmt3a^R878H/WT^ mice compared with the Dnmt3a^WT/WT^ controls. The mean ± SD of minimum n = 3 independent experiments is displayed, representative images shown *p ≤ 0.05, **p ≤ 0.01 (Student’s T-test)

We explored the metabolic difference in the serum of Dnmt3a^R878H/WT^ mice compared with the Dnmt3a^WT/WT^ mice. UHPLC-HRMS/MS detection on the serum metabolites of Dnmt3a^R878H/WT^ and Dnmt3a^WT/WT^ mice showed 55 differential metabolites (Fig. 1A). KEGG enrichment revealed that the most notable changes belonged to the metabolism of nicotinate and NAM (Fig. 1B), as reflected in the decreased levels of NAM and 1-methylnicotinamide in the serum of Dnmt3a^R878H/WT^ mice (Fig. 1C). The downstream metabolites of NAM including 1-methylnicotinamide and adenosine 3'-monophosphate (AMP) were elevated in the Gr1^+^ cells (leukemic cells) of Dnmt3a^R878H/WT^ mice (Fig. 1D). GO analysis was performed on the RNA-seq data from leukemic cells of the Dnmt3a^R878H/WT^ mice, and the result indicated significant changes in the regulation of NMN and NAD metabolic pathways (Fig. 1E). The transcription and translation levels of Nampt, an important rate-limiting enzyme of NAM metabolism and NAD synthesis, were upregulated in Gr1^+^ cells (leukemic cells) of Dnmt3a^R878H/WT^ mice (Fig. 1F, G). MeDIP-seq was performed on the Gr1^+^ cells of the Dnmt3a^R878H/WT^ mice and showed that Nampt was hypomethylated at the gene body region (Fig. 1H). These results indicated that the Dnmt3a-R878H mutation upregulated Nampt expression by regulating DNA methylation.

### DNMT3A mutation leads to aberrant expression of NAMPT to produce more NAD+/NADH

**Fig. 2:**
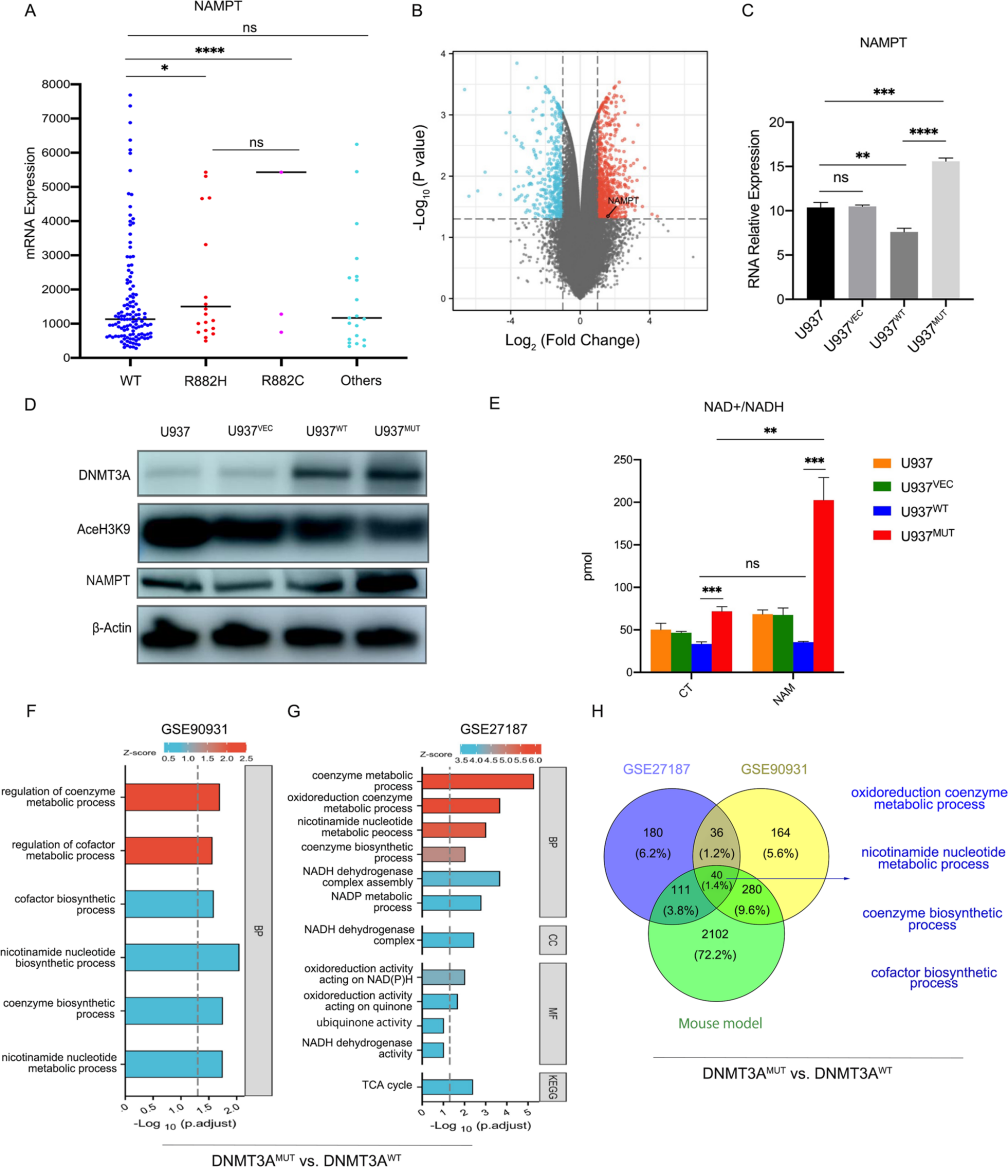
The DNMT3A mutation results in high expression of NAMPT and aberrant NAM-NAD metabolism. **A** NAMPT expression levels in patient samples with DNMT3A wild-type, DNMT3A-R882H mutation, DNMT3A-R882C mutation and other mutation sites in AML (n = 200) in the TCGA database. **B** The volcano map showed the higher expression of NAMPT in U937^MUT^ compared with the U937^WT^ and U937^VEC^. **C** Q-RT-PCR showed the mRNA expression level of NAMPT in U937, U937^VEC^, U937^WT^ and U937^MUT^. **D** Immunoblotting showed the protein level of NAMPT in U937, U937^VEC^, U937^WT^ and U937^MUT^. **E** The content of original intracellular NAD + /NADH and the NAD + /NADH converted from NAM with NAMPT in the U937, U937^VEC^, U937^WT^ and U937^MUT^. **F** GO enrichment analysis of the differential genes in U937^MUT^ vs. U937^WT^. **G** GO enrichment analysis of the differential genes in leukemic cells in patient samples with the DNMT3A R882 mutation vs. the DNMT3A wild-type. **H** The Venn diagram showed 40 common abnormal metabolic pathways in GSE27187, GSE90931 and Dnmt3a^R878H/WT^ mouse model. The mean ± SD of minimum n = 3 independent experiments is displayed, representative images shown *p ≤ 0.05, **p ≤ 0.01, ***p ≤ 0.001 and ****p ≤ 0.0001 (Student’s T-test)

Analysis of the TCGA AML database showed that the mRNA expression level of NAMPT was significantly increased in patient samples with the DNMT3A mutation compared to the DNMT3A wild-type (Fig. 2A). The analysis of RNA-seq data from GSE90931 showed that U937 cells carrying the DNMT3A mutation expressed higher NAMPT (Fig. 2B). We constructed U937 cell lines carrying DNMT3A R882H mutation (U937^MUT^), wild-type DNMT3A (U937^WT^) and the vector (U937^VEC^), respectively. Q-RT-PCR and immunoblotting showed that both the mRNA and protein levels of NAMPT were significantly elevated in the U937^MUT^ cells compared to the U937^WT^ (Fig. 2C, D). Meanwhile, the U937^MUT^ had more NAD+/NADH content and stronger ability to metabolize NAM to NAD+/NADH (Fig. 2E). GO analysis of differentially expressed genes showed significant enrichment of pathways involved in the nicotinamide nucleotide biosynthetic process and coenzyme metabolic process in both patient samples (GSE27187) and U937 cell line (GSE90931) harboring the DNMT3A mutation (Fig. 2F, G). Moreover, NMN metabolic process and coenzyme/cofactor biosynthetic process pathways were highly enriched in the Dnmt3a^R878H/WT^ mice vs. the Dnmt3a^WT/WT^ mice. In datasets GSE90931 and GSE27187, these metabolic pathways were also significantly enriched in the mutation-carrying samples compared with the wild-type samples (Fig. 2H).

### The aberrant NAM metabolism caused by the DNMT3A mutation promotes cell cycle progression

**Fig. 3:**
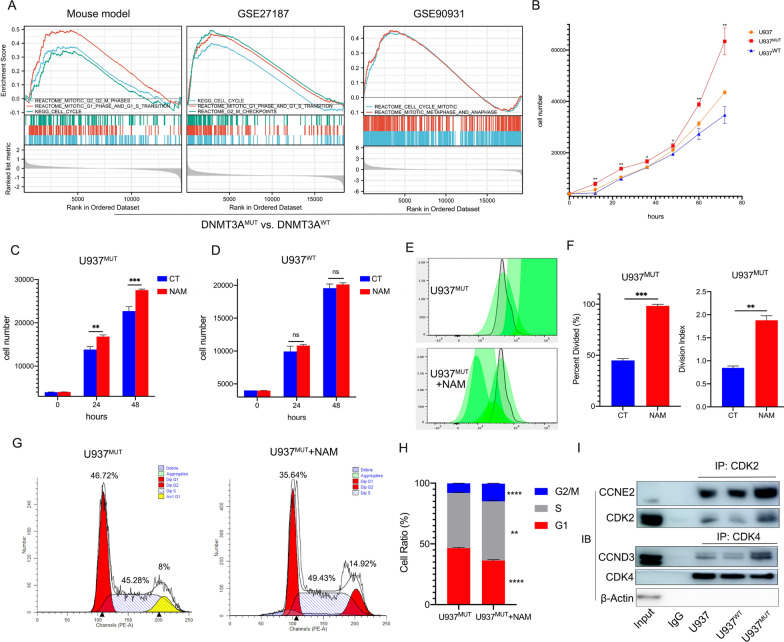
The DNMT3A mutation accelerates cell cycle through NAM metabolic reprogramming. **A** GSEA enrichment of differential genes in the Dnmt3a^R878H/WT^ mouse model, human AML-M5 samples with the DNMT3A R882 mutation and U937 cell lines with the DNMT3A-R882H mutation compared with the wild type. **B** Proliferation velocity of U937, U937^MUT^ and U937^WT^ cell lines. **C**, **D** The changes of cell number of the U937^MUT^ and U937^WT^ cell lines with the treatment of NAM. **E** Cell division of U937^MUT^ cell line indicated by CFSE after NAM treatment. **F** Percent divided and division index of U937^MUT^ after NAM treatment. **G**, **H** Cell cycle changes of the U937^MUT^ cell line after NAM treatment indicated by PI (propidium iodide). **I** Different binding ability between CDK2-CCNE2 and CDK4-CCND3 in U937^WT^ and U937^MUT^. The mean ± SD of minimum n = 3 independent experiments is displayed, representative images shown *p ≤ 0.05, **p ≤ 0.01, ***p ≤ 0.001 and ****p ≤ 0.0001 (Student’s T-test)

GSEA (Gene Set Enrichment Analysis) of RNA-seq data showed statistically significant enrichment of the cell cycle and mitotic signaling pathways in Gr1^+^ cells of Dnmt3a^R878H/WT^ mice compared with Dnmt3a^WT/WT^ mice. Human samples (GSE27187) and U937 cell line carrying this mutation showed the similar results (Fig. 3A). U937^MUT^ cell line showed a higher proliferation efficiency compared with U937^WT^ (Fig. 3B). After adding 50 µm NAM to the medium, we found that NAM could accelerate the proliferation efficiency of U937^MUT^, which was not observed in U937^WT^ (Fig. 3C, D). NAM increased the percent divided and division index of U937^MUT^ cells (Fig. 3E, F). And NAM made more U937^MUT^ cells enter S phase and G2 phase (Fig. 3G, H). Co-immunoprecipitation experiments revealed that the DNMT3A mutation can enhance the binding ability of CDK2-CCNE2 and CDK4-CCND3 (Fig. 3I).

### Inhibition of NAMPT induces depolymerization of Cyclins-CDKs through SIRT6

**Fig. 4:**
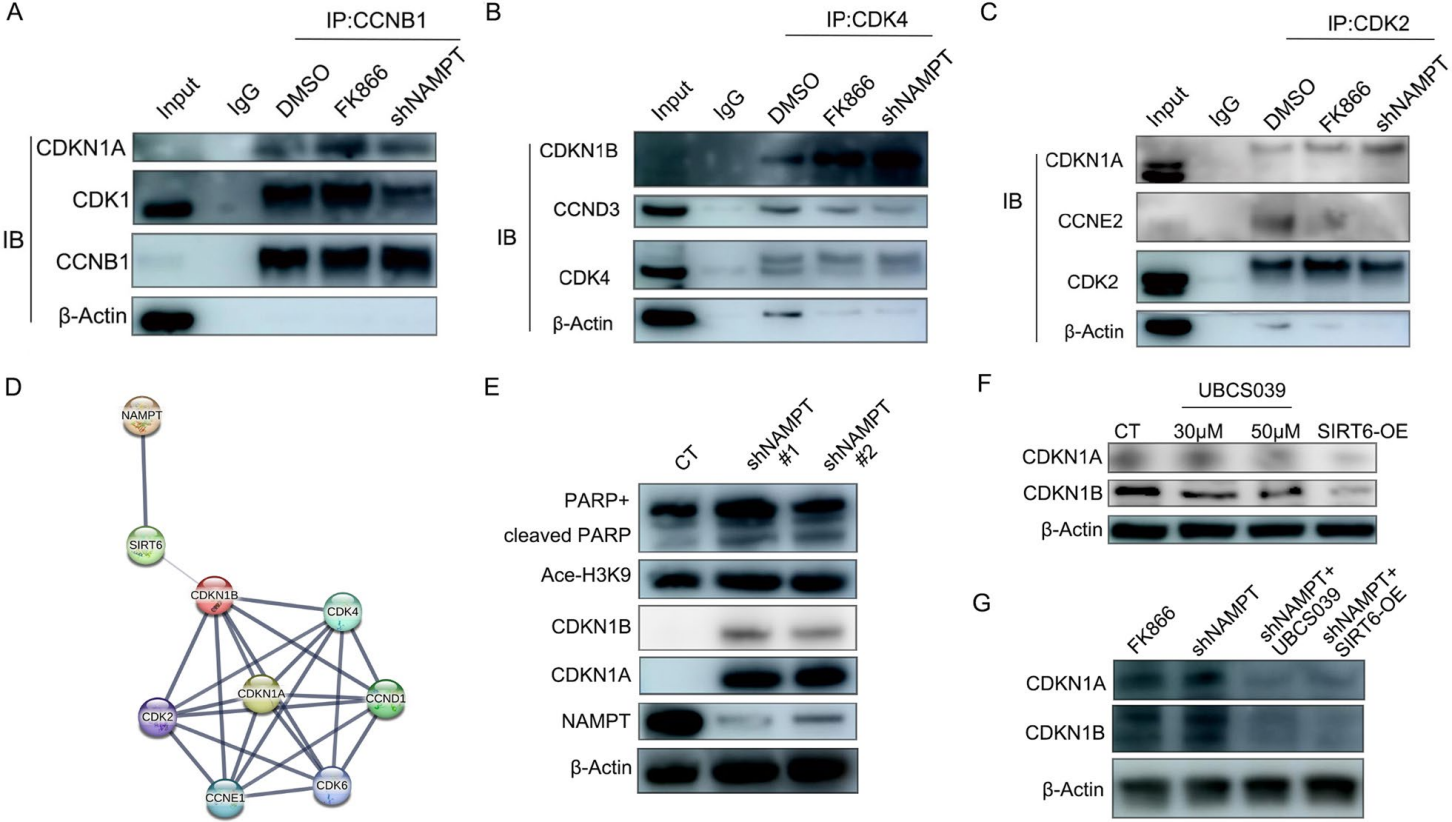
Inhibition of NAMPT can induce the depolymerization of Cyclins-CDKs through SIRT6. **A–C** Co-immunoprecipitation of bindings of Cyclins-CDKs and the corresponding CDKN1A and CDKN1B in OCI-AML3 in which NAMPT was pharmacological or genetic inhibited. **D** The prediction of interaction between NAMPT and cell-cycle related proteins using the String database. **E** After NAMPT knockdown, the protein levels of CDKN1A/CDKN1B and Ace-H3K9 were detected by the immunoblotting. **F** The immunoblotting of the CDKN1A and CDKN1B levels in OCI-AML3 after treatment with UBCS039 or overexpression of SIRT6. **G** OCI-AML3 cells were genetically or pharmacologically inhibited, and the shNAMPT-OCI-AML3 cells were treated with UBCS039 or overexpressed SIRT6. The immunoblotting of CDKN1A and CDKN1B was then analyzed

To determine whether NAMPT could regulate the binding ability of Cyclins-CDKs, co-immunoprecipitation experiments were performed to analyze the effects of pharmacological or genetic inhibition of NAMPT on OCI-AML3 harboring DNMT3A R882C mutation. The results showed that after NAMPT inhibition or knockdown, the formation ability of the CDK1-CCNB1, CDK4-CCND3, and CDK2-CCNE2 complexes deteriorated, whereas the binding ability of CDKN1A/CDKN1B to the corresponding Cyclins-CDKs was enhanced (Fig. 4A–C). The analysis using the String database showed that NAMPT may affect the binding of CDKN1A/CDKN1B to Cyclins-CDKs complexes through SIRT6 (Fig. 4D). Immunoblotting showed that the deacetylation function on Ace-H3K9 of SIRT6 was affected by NAMPT knockdown, accompanied with a marked increase in CDKN1A/CDKN1B protein levels (Fig. 4E). Stimulation of OCI-AML3 with the SIRT6 agonist UBCS039 or overexpression of SIRT6 on OCI-AML3 can decrease the protein levels of CDKN1A/CDKN1B (Fig. 4F). Moreover, it was observed that the increased expression of CDKN1A and CDKN1B in shNAMPT-OCI-AML3 can be inverted by UBCS039 or overexpression of SIRT6 (Fig. 4G).

### DNMT3A mutation endows cells with sensitivity to NAMPT inhibitor, and inhibition of NAMPT causes cell apoptosis, cycle arrest and differentiation

**Fig. 5:**
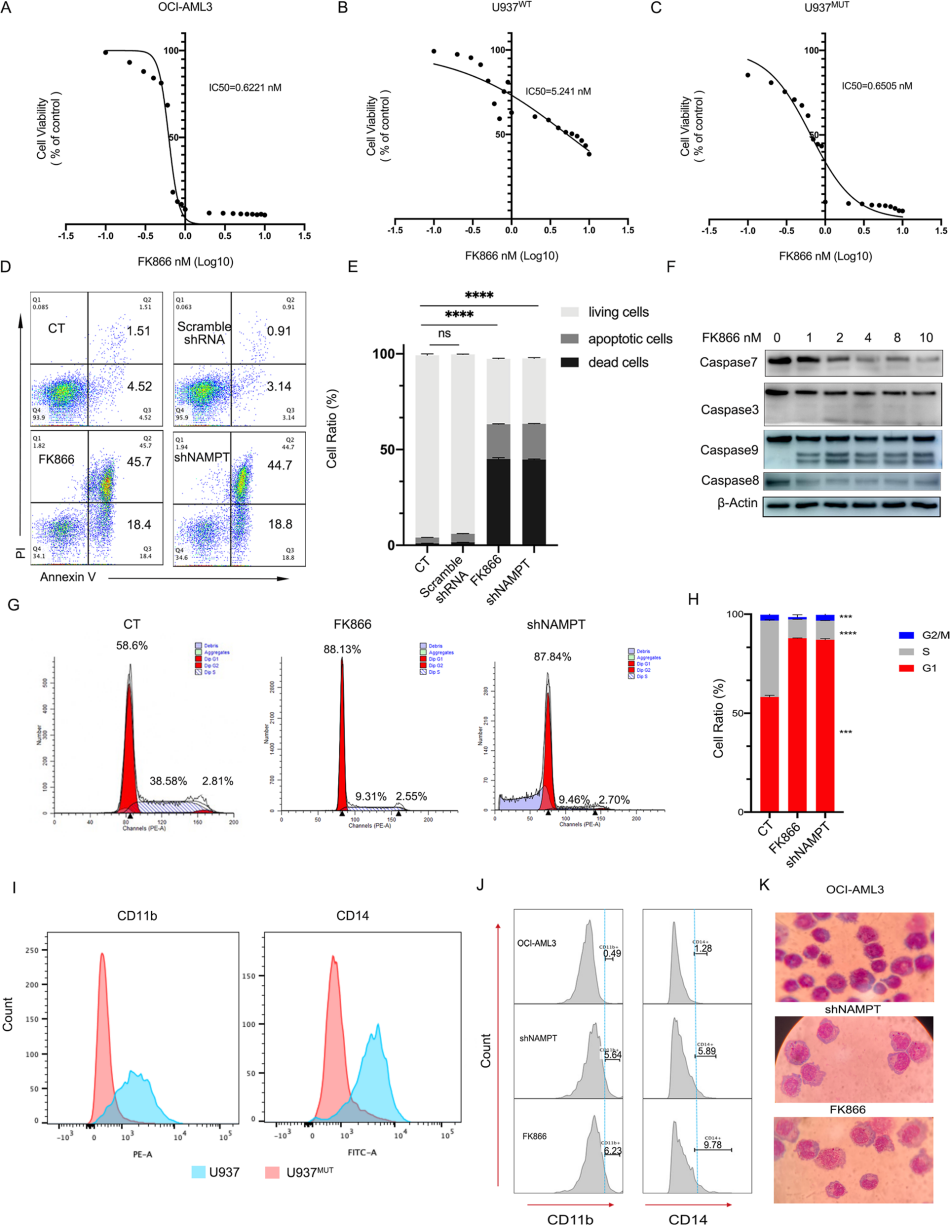
Sensitivity of DNMT3A mutant cells to FK866 and the effects of inhibition on NAMPT. **A–C** Cell viability of OCI-AML3, U937^WT^ and U937^MUT^ after treatment with FK866 for 48 h and the IC50 of each was calculated. **D**, **E** Cell apoptosis after shNAMPT or NAMPT inhibition using FK866. **F** Changes of apoptosis-initiating proteins after NAMPT inhibition using FK866. **G**, **H** Cell cycle arrest after knockdown (shNAMPT) or inhibition of NAMPT (FK866). **I** Changes of cell differentiation-related markers CD11b and CD14 after introduction of the DNMT3A-R882H mutation to U937 cell line. **J**, **K** Changes of CD11b and CD14 and cell morphology after knockdown or inhibition of NAMPT. The mean ± SD of minimum n = 3 independent experiments is displayed, representative images shown *p ≤ 0.05, **p ≤ 0.01, ***p ≤ 0.001 and ****p ≤ 0.0001 (Student’s T-test)

To investigate whether high NAMPT expression due to the DNMT3A mutation makes leukemia cells more sensitive to NAMPT inhibition, OCI-AML3 and U937^WT^/U937^MUT^ cell lines were treated with the NAMPT inhibitor FK866. The results showed that the DNMT3A mutation conferred cells sensitivity to FK866 with much lower IC50 (Fig. 5A–C). Moreover, pharmacological or genetic inhibition of NAMPT induced apparent apoptosis (Fig. 5D, E), which was confirmed by the activation of apoptosis-initiating proteins including caspase8, 9, 3 and 7 (Fig. 5F). It was observed that inhibition of NAMPT reduced the proportion of cells entering S and G2 phase, and the most cells were arrested in G1 phase (Fig. 5G, H). In terms of arresting cell differentiation, the introduction of the DNMT3A mutation reduced the expression of CD11b and CD14 in U937^MUT^ (Fig. 5I). And we found that inhibition of NAMPT increased the expression of CD11b and CD14 in OCI-AML3 cells originally carrying the DNMT3A mutation with the morphology of cells becoming more mature (Fig. 5J, K).

### Inhibition of NAMPT significantly prolongs lifespan and reduces splenic infiltration of tumor cells in tumor-bearing mice

**Fig. 6:**
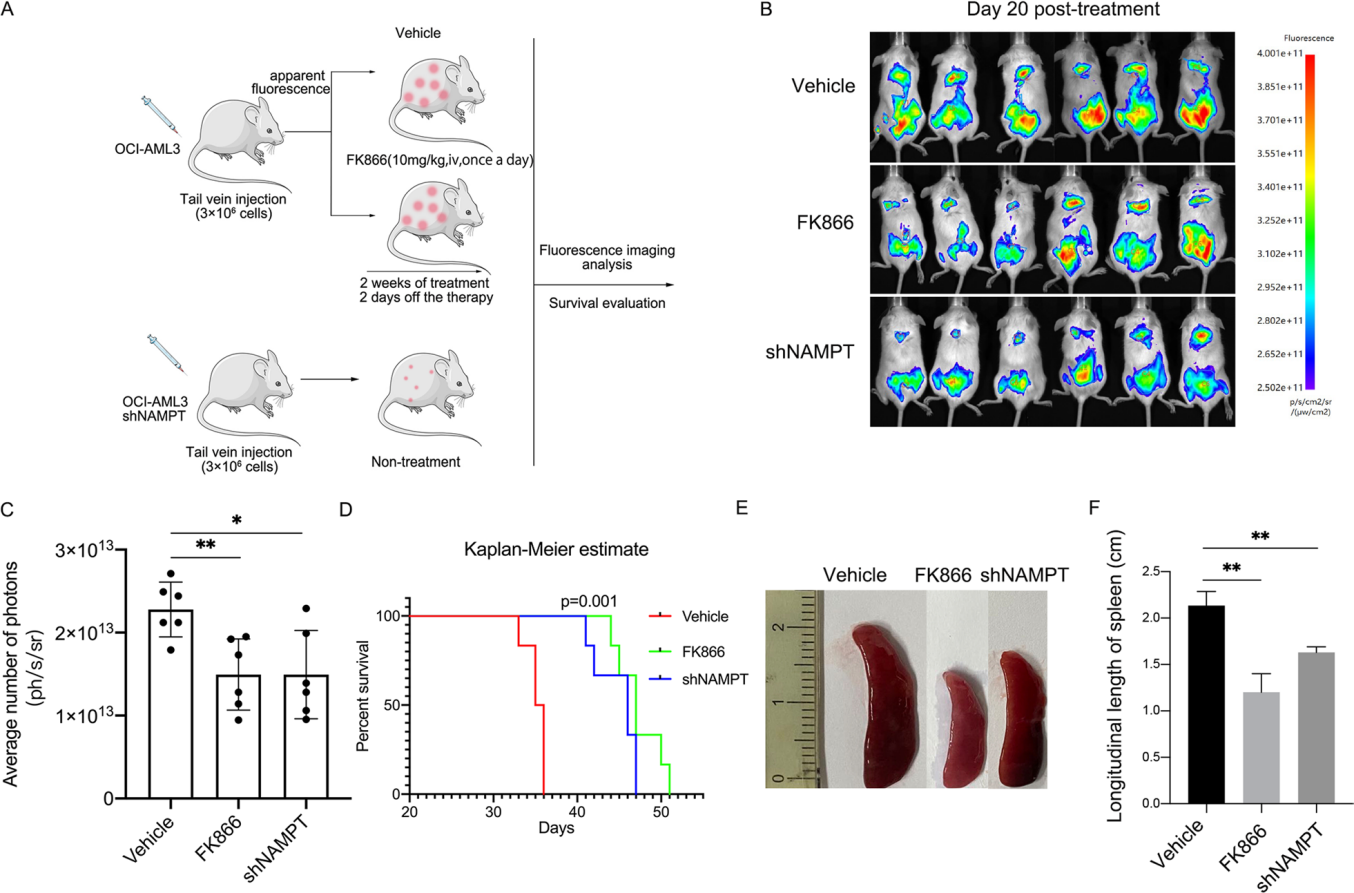
Intervention of NAMPT prolongs the survival of tumor-bearing mice and reduces splenic infiltration. **A** Schematic diagram of the animal experiment. **B**, **C** Tumor fluorescence imaging and mean photon counts of three groups of mice after treatment or non-treatment for 20 days. **D** Kaplan–Meier survival curve of the three groups of mice. **E** Splenic infiltration of tumors of the three groups of mice. F The statistical diagram depicts the length of the longitudinal axis of the spleen in the indicated three groups of mice (n = 5 for each group). *p ≤ 0.05, **p ≤ 0.01 (Student’s T-test)

NOG mice were selected for in vivo experiments to further explore the effect of inhibiting NAMPT on tumor. 3 × 10^6^ OCI-AML3 or shNAMPT-OCI-AML3 cells was infused into mice via tail vein (n = 6 for each group). In vivo fluorescence imaging showed obvious tumor cell fluorescence in mice 3 days post-infusion, which were subsequently treated with FK866 or vehicle (Fig. 6A). FK866 treatment or knockdown of NAMPT resulted in slower tumor growth (Fig. 6B, C) and longer overall survival in tumor-bearing mice compared with the controls (Fig. 6D). Furthermore, inhibition or NAMPT knockdown significantly reduced splenic infiltration of tumor cells (Fig. 6E).

## Discussion

The occurrence and development of AML is a complex and multi-step process involving a series of genetic abnormalities [31–34]. One of the main categories is gene mutations related to epigenetic regulation, including DNMT3A, IDH1, IDH2, TET2, MLL, ASXL1, EZH2, UTX and others. Epigenetics and metabolism can interact and influence each other. For example, both histone and DNA methylation require S-adenosyl methionine [35], and S-adenosylhomocysteine produced by these reactions in turn inhibits the activity of DNA methylation Transferase (DNMT) and histone methyltransferase [36]. About 8–19% of AML patients have isocitrate dehydrogenase-2 (IDH2) mutation, which can significantly increase the metabolite 2-HG [37]. 2-HG can cause DNA and histone hypermethylation, and then up-regulate the expression of downstream target genes that inhibit cell differentiation, resulting in blocked cell differentiation and tumorigenesis [38]. Targeting IDH mutant enzymes can reduce the production of oncogenic metabolites, reverse epigenetic regulatory disturbances, and alleviate cell differentiation arrest [39].

DNMT3A is responsible for the modification of DNA de novo methylation and is frequently mutated in AML-M4 and AML-M5 (FAB subtype). It is generally believed that DNMT3A mutation is a driver mutation in the development of leukemia as an early event. However, the metabolic effects of the DNMT3A mutation on AML, especially the in vivo data, remains unclear. In our study, we constructed Dnmt3a^R878H/WT^ conditional knock in mouse model as previously described [30], and detected the differential metabolites of serum from the knock in mice and the WT controls by mass spectrometry technology. It was found that the Dnmt3a mutation could lead to abnormal NAM metabolism by upregulating the key enzyme Nampt through hypomethylation at the gene body region. There are several evidences that prove methylation level at gene body region could regulate gene expression [40, 41]. We hypothesized that the hypomethylation at the gene body region of Nampt could potentially impact the accessibility of chromatin and consequently lead to an increase in the transcriptional activity of Nampt. By constructing U937 cell line with DNMT3A R882H mutation, we found that DNMT3A mutation can indeed lead to increased expression of NAMPT. Analysis of the TCGA database also revealed that samples from AML patients carrying DNMT3A mutation had a higher expression level of NAMPT. Analysis of the Oncomine database showed that 72% of AML with high expression of NAMPT belonged to AML-M4 or AML-M5 (Additional file 2: Table S2), which are closely related with DNMT3A mutations.

Based on the higher demand for ATP and the shorter half-life of NAD in cancer cells, many studies have focused on the abnormal expression of rate-limiting enzymes including NAMPT and NAPRT in tumor. Epigenetic silencing of the frequently mutated PPM1D-driven NAPRT confers sensitivity to NAMPT inhibition in pediatric gliomas [42]. Also, IDH1-mutated glioma types are extremely sensitive to NAMPT loss [43]. However, the effect of metabolic reprogramming caused by abnormal expression of NAMPT on the biological function of tumor cells is lack of research. By adding NAM to the medium and detecting the intracellular NAD content, we found that the ability of the U937^MUT^ to convert NAM into NAD was much higher than that of the U937^WT^, and this behavior was consistent with the high expression of NAMPT in the former. Integrated metabolomics, transcriptome and enrichment analysis revealed that DNMT3A mutation can lead to the upregulation of NAM, NMN and NAD metabolic pathways and coenzyme biosynthetic process. Moreover, DNMT3A mutation conferred sensitivity of OCI-AML3 and U937^MUT^ to NAMPT inhibition. The reason may be that the DNMT3A-mutated cells prefer the NAMPT-dependent NAD salvage synthesis pathway.

We have previously defined Gr1^+^ cells from the Lin^−^Sca1^+^c-Kit^+^ cells of the Dnmt3a^R878H/WT^ mouse model as leukemic cells responsible for the unique AML phenotype. Through transcriptome sequencing of Gr1^+^ cells, we found that many proliferation-related genes such as Pcna and Cdk family members were overexpressed, and CDK1 overexpression promoted the occurrence of leukemia by regulating the interaction between EZH2 and DNMT3A [44]. In this study, GSEA of mice, cell lines, and human samples showed that the DNMT3A mutation upregulated cell cycle and mitotic phase transition pathways, which was consistent with previous findings. Co-immunoprecipitation experiments showed that CDK2-CCNE2 and CDK4-CCND3 were more bound in U937^MUT^ than that in U937^WT^ cells. Furthermore, we found that the growth rate of U937^MUT^ cell line was higher than that of U937^WT^, and the addition of NAM accelerated the growth rate and division rate of U937^MUT^ cells, and made more cells enter S phase and G2 phase. Inhibition of NAMPT can induce cell cycle arrest by depolymerizing Cyclins-CDKs. In the in vivo experiment, pharmacological or genetic inhibition of NAMPT significantly prolonged the overall survival of the tumor bearing mice and reduced the splenic infiltration of tumor cells.

In this study, we mainly focused on the metabolic reprogramming of cells by regulating the expression of intracellular NAMPT (iNAMPT) caused by DNMT3A mutation. However, the possible impact of the DNMT3A mutation on the secretion and delivery of extracellular NAMPT (eNAMPT) need to be further explored. Studies have shown that eNAMPT is carried by extracellular vesicles and participates in systemic circulation in mice and humans. Levels of eNAMPT (particularly serum eNAMPT) have also been shown to increase in cancer and possibly contribute to angiogenesis and induction of an inflammatory cancer microenvironment and finally to poor prognosis [45]. In leukemia, eNAMPT functions in promoting an immunosuppressive and pro-tumor microenvironment in chronic lymphocytic leukemia and it is important for the differentiation of monocytes into tumor-supporting M2 macrophages [46, 47]. The link between the DNMT3A mutation and eNAMPT, and the interaction between iNAMPT and eNAMPT in tumors carrying this mutation, need to be further investigated, which will help us uncover the potential of combining intracellular NAMPT inhibitors with extracellular NAMPT neutralizing antibodies as a therapeutic strategy.

## Conclusions

**Fig. 7:**
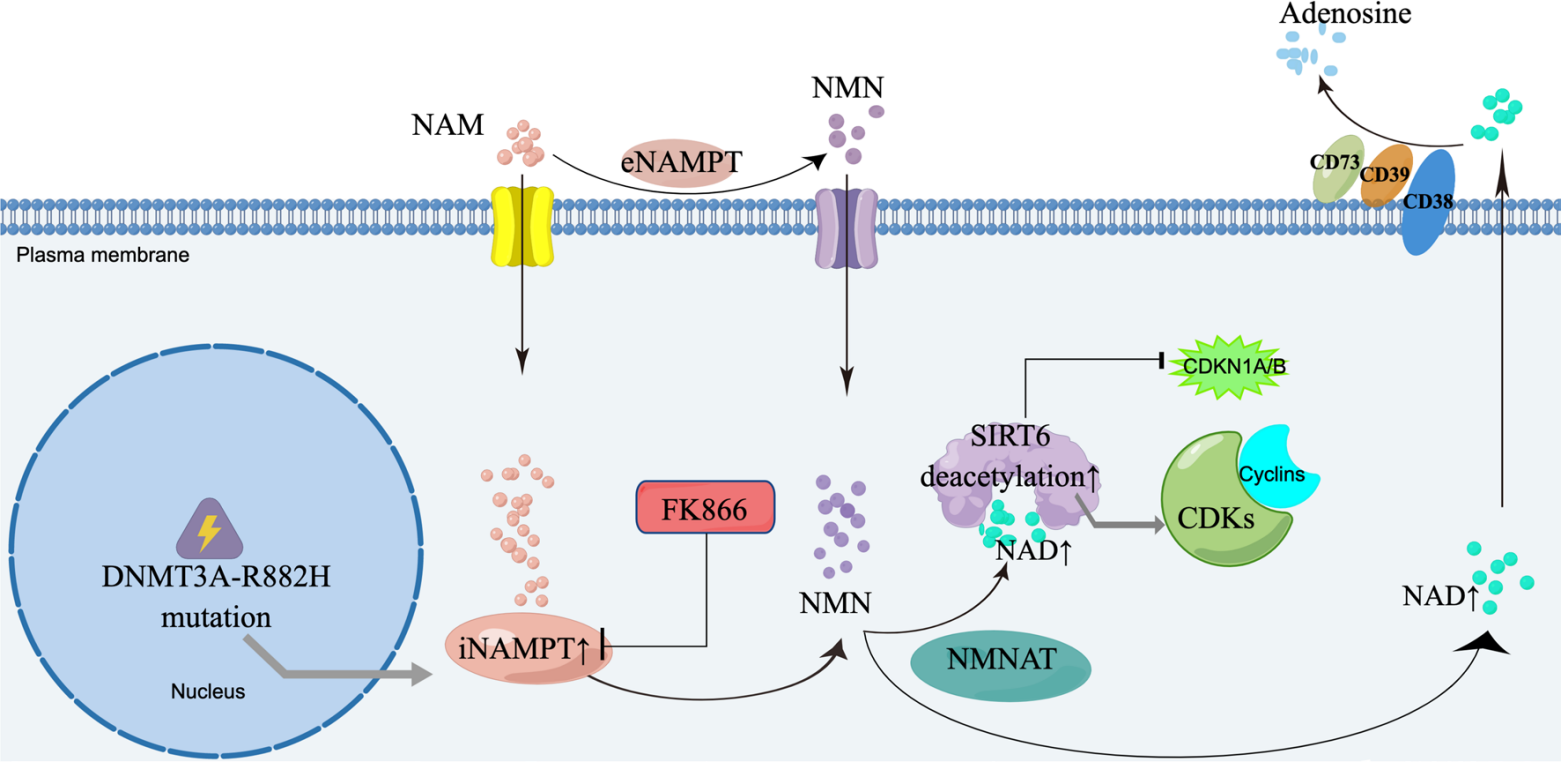
Schematic diagram showing the abnormal NAM-NAD metabolism caused by DNMT3A R882H mutation and the effect of intervention on it

In this study, we showed that the NAM-NAD metabolic reprogramming and high NAMPT expression induced by DNMT3A mutation further promoted the proliferation of leukemia cells. The intervention of the key enzyme NAMPT depolymerized the Cyclins-CDKs complexes and promoted the binding of CDK inhibitors to these complexes (Fig. 7). Leukemia cells with high NAMPT expression are more sensitive to the NAMPT inhibitor. In vivo experiment also showed that inhibition of NAMPT prolonged survival and reduced the splenic infiltration of tumor cells in mice. These results provided a potential strategy at the metabolic level for targeted therapy of AML patients with DNMT3A mutations.

## Supplementary Information

**Additional file 1: Table S1.** Primer sequences used in Q-RT-PCR.

**Additional file 2: Table S2.** The mRNA expression level of NAMPT in AML obtained from the Oncomine database.

## Data Availability

The data sets used in this study are available from the corresponding author on reasonable request.

## Abbreviations

DNMT3A: DNA methyltransferase 3A
NAM: Nicotinamide
NAD: Nicotinamide adenine dinucleotide
AML: Acute myeloid leukemia
NAMPT: Nicotinamide phosphoribosyltransferase
NMN: Nicotinamide mononucleotide
NMNAT: Nicotinamide mononucleotide adenylyltransferase
2-HG: 2-Hydroxyglutarate
Q-RT-PCR: Quantitative reverse transcription-polymerase chain reaction
CDK: Cyclin-dependent kinase
CDKI: Cyclin-dependent kinase inhibitor
UHPLC-HRMS/MS: Ultra-High Performance Liquid Chromatography–High Resolution Tandem Mass Spectrometry
MeDIP-seq: Methylated DNA Immunoprecipitation Sequencing
HSC: Hematopoietic stem cells
LSC: Leukemia stem cells
IDH2: Dehydrogenase-2
