# Supplementary material for: DNMT3A mutation promotes leukemia development through NAM-NAD metabolic reprogramming
Source: J Transl Med. 2023 Jul 18;21:481. doi: 10.1186/s12967-023-04323-z (PMC10355022; doi:10.1186/s12967-023-04323-z)
Supplement: Supplementary file 1 — Additional file 1: Table S1. Primer sequences used in Q-RT-PCR. [file 12967_2023_4323_MOESM1_ESM.docx]

Table S1: Sequences of human and mouse primers for quantitative RT-PCR.

| Gene name | Forward | Reverse |
| --- | --- | --- |
| NAMPT | AGCAGAACACAGTACCATAACA | CCCATATTTTCTCACACGCATT |
| Nampt | CTGTGTCTGTGGTCAGCGATAGC | GGTCTGATGATTAGTGGTGCCTCTG |
